# Effectiveness of interventions to promote pesticide safety and reduce pesticide exposure in agricultural health studies: A systematic review

**DOI:** 10.1371/journal.pone.0245766

**Published:** 2021-01-26

**Authors:** Maryam Afshari, Akram Karimi-Shahanjarini, Sahar Khoshravesh, Fereshteh Besharati

**Affiliations:** 1 Department of Public Health, Hamadan University of Medical Sciences, Hamadan, Iran; 2 Social Determinants of Health Research Center, Hamadan University of Medical Sciences, Hamadan, Iran; 3 Department of Nursing, Zeynab (P.B.U.H) School of Nursing and Midwifery, Guilan University of Medical Sciences, Rasht, Iran; Zagazig University, EGYPT

## Abstract

**Objective:**

There is a relationship between pesticide exposure and farmworkers’ health. Well-conducted evaluations can provide an insight into how to develop and implement more effective interventions to prevent farmers and farmworkers’ exposure to pesticides. This review aimed to summarize the literature on the effectiveness of interventions to promote pesticide safety and reduce pesticide exposure among farmers and farmworkers.

**Methods:**

A comprehensive search on PubMed, Embase, ISI Web of Science, Scopus, Science Direct, Agricola, NIOSHTIC, and Agris databases was performed to identify relevant studies published from 2000 to 2019. Randomized controlled trials (RCTs) and quasi-experimental studies assessing the effectiveness of interventions on a variety of outcomes related to pesticide exposure were considered. The searches were restricted to articles written in English. The methodological quality of included reviews was appraised using the Effective Public Health Practice Project quality assessment tool (EPHPP).

**Results:**

The initial search led to 47912 records, 31 studies of which including nine RCTs and twenty-two quasi-experimental studies met the criteria. The majority of the included studies focused on the educational/ behavioral approach. The studies that applied this approach were effective in improving the participants’ knowledge and attitude; however, these interventions were less effective in terms of making changes in participants’ behaviors and their risk of exposure to toxic pesticides. Multifaceted interventions were moderately effective in terms of improving farmers’ and farmworkers’ behaviors and reduction in exposure to toxic pesticides. We did not find any studies that had evaluated the effectiveness of engineering/technological, and legislation/enforcement interventions.

**Conclusions:**

Although the majority of studies were based on an educational/behavioral approach and did not assess the effect of interventions on objective measures, the results of this review highlight the significant effectiveness of educational programs and some potential key elements of these interventions. These findings may inform policymakers to develop interventions to reduce pesticide exposure among farmers and farmworkers.

## Introduction

Agriculture is a pillar of development and achieving the goal of sustainable agriculture is one of the most important goals and responsibilities of each country. As reported by the World Bank (2010), about 20% of workers were employed in agriculture worldwide [1]. Regardless of working conditions, due to exposure to hazardous factors, agriculture has been ranked as one of the most hazardous industries [2].

The use of pesticides may cause serious health problems in farmers and farmworkers [3]. Pesticide use is dramatically increasing worldwide. Optimum and safe use of pesticides and chemical fertilizers are essential to save agricultural products from poisonous contamination and farmers’ health [4]. However, along with the advantages of pesticides, there are also threats such as environmental pollution and acute pesticide poisonings due to improper and unsafe handling [5].

Several factors associated with farmers’ pesticide exposure have been reported in previous investigations. Some of these factors include using banned or restricted pesticides, the lack use of appropriate equipment along with taking protective activities that are needed during pesticide handling [6], label non-compliance, improper spraying or protective equipment, unsafe pesticide disposal [7], insufficient knowledge of the law, limitations of environmental standards [8], and lack of training [9].

Existing evidence on farmers and farmworkers’ health highlights a research gap in this regard. Particularly, the effectiveness of varied interventions developed to reduce agricultural injuries is not well documented [10]. The well-conducted evaluations would increase our knowledge of the prevention of farmers’ pesticide exposure by documenting what works. These efforts could be useful in identifying the most cost-effective programs in this area.

We identified two recent reviews which focused on the effectiveness of interventions to reduce pesticide exposure among farmers. Both of these reviews focused only on the effectiveness of educational interventions [11, 12]. So, there is limited evidence to support the effectiveness of several types of interventions that are used to improve knowledge, attitude, beliefs, practice/ behavior, and exposure monitoring methods related to reducing pesticide exposure among farmers and farmworkers. Also, neither review assessed the methodological quality of the included studies. This systematic review was conducted to identify and describe the effectiveness of various types of interventions that have been designed to promote pesticide safety and reduce exposure to pesticides amongst the farmers and farmworkers.

## Methods

We followed PRISMA (Preferred Reporting Items for Systematic Reviews and Meta-analyses) as a guideline to document and report our search and screening process. Applying this guideline increases the transparency and quality of reporting [13].

### Search strategies

We used search terms that covered the concepts of pesticide exposure, farmers and farmworkers, and study design to identify studies in the electronic databases. Search strategies were obtained from the previous systematic reviews [10–12, 14]. We then modified it to fit the features of the databases. All databases were searched up to August 2019.

We searched the following electronic literature databases: PubMed, Agricola, Embase, ISI Web of Science, Science Direct, Scopus, NIOSHTIC-2, Agris. We also tracked the citation of both forward and backward reference lists of included articles and relevant reviews were checked for all included studies.

The search strategy for PubMed is described in S1 Appendix.

### Inclusion criteria

We included studies published in English peer-reviewed journals and had the following criteria:

#### Study population

Our focus was on farmers and farmworkers, although we did not exclude interventions that had some components for other groups including farmers and farmworkers’ families.

#### Types of studies

We included studies that were randomized controlled interventions (RCT), pretest/ post-test interventions (PPI) or controlled pretest/post-test interventions (cPPI). Descriptive, qualitative, review, systematic review, and meta-analysis studies to reduce exposure to pesticides in agriculture were excluded.

#### Types of interventions

We included interventions at the national, regional, organizational, community, or individual level deliberately designed to reduce exposure to pesticides or poisoning.

#### Types of outcomes

We selected studies in which the primary outcome measure was a subjective (e.g., knowledge/ awareness, attitude, risk perception, and reporting personal protective equipment use) or objective (i.e., measures such as cholinesterase determined by biological monitoring or exposure monitoring) measure of pesticide exposure. We did not consider studies in which the evaluation of outcomes was conducted using qualitative data. In addition, we considered the studies the outcomes of which emphasized only pesticide exposure in three aspects including knowledge, attitude and beliefs, practice or behavior, and exposure monitoring methods.

### Study selection

Titles and abstracts of studies retrieved by electronic searching were independently screened for eligibility by the three co-authors (M.A, F.B, and S.K). Any discrepancies between the reviewers were resolved through discussion with a fourth review (A. K-Sh). When it was not possible to assess the eligibility of studies based on the title and abstract, full-text versions were obtained.

### Data extraction

Data were extracted independently by two authors (S.K and M.A). A data extraction template was developed by the research team based on the goals of the study that included the title and author(s), place of study, objective of the study, sample size, characteristics of participants, intervention package (including strategies used, target outcome(s), use of behavioral theory, follow-up duration, measurement tools used to evaluate the effectiveness of intervention), and change in target outcome(s). Any disagreements were resolved by a fourth reviewer (A. K-Sh). Details of the included studies are reported in Tables 1 to 3. Due to the differences in interventions and target outcomes, no meta-analysis was attempted on the included studies.

**Table 1:** Effectiveness of randomised controlled trials studies to reduce pesticide exposures.

Authors/ Country	Objective of the study	Participants	Intervention focus/	Theoretical framework	Outcomes		
			Intervention approach and strategies/ Measures		Knowledge, attitude and beliefs*	Practice / behavior**	Exposure monitoring methods***
Farahat et al. (2009)/ Egypt	To assess the effectiveness of educational interventions directed towards farming families to protect their children from the hazards of pesticide exposure	N = 297	• Farmers and their families	None specified	Non -significant differences in knowledge between two groups at 2-week and 1- month follow up (P> 0.05)	Non-significant differences in practice between two groups at 2-week and 1- month follow up (P> 0.05)	
		I: n = 132	• Educational/ behavioral intervention approach:				
		C: n = 165					
			• I: Three educational sessions for parents in videotape group				
			• C: Three educational sessions for parents in lecture group				
			Measures: baseline, 2 –week and 1- month follow—up				
Thompson et al. (2008)/ United States of America	To evaluate the effectiveness of a community intervention to reduce pesticide exposure among farmworkers and their children.	N = 529	• Farmworkers and their families	None specified			Non-significant differences in urinary pesticide metabolite concentrations among adult and child between two groups
		I: n = 245	• Multi-faceted interventions:				
		C: n = 284	• I: Intervention at community level (provided information in settings such as health fairs) + intervention at organizational level (raising knowledge in schools and churches as well as offering packets of equipment such as detergent and clothes sorting bags) + intervention at the small-group level (holding home health party and discussing a specific pesticide topic) +interventions at the individual level (raising knowledge and distributing laundry kits and shower kits)				
			• C: No intervention				
			• Measures: Baseline and 4- year follow-up				
Strong et al. (2009)/ United States of America	To evaluate the effectiveness of a community intervention in promoting the adoption of behaviors to reduce pesticide exposure in farmworker households	N = 529	• Farmworkers and their families	None specified		Significant difference only in one of six assessed behaviors between two groups (P = 0.004)	
		I: n = 245	• Multi-faceted interventions:				
		C: n = 284	• I: Intervention at community level (provided information in settings such as health fairs) + intervention at organizational level (raising knowledge in schools and churches as well as offering packets of equipment such as detergent and clothes sorting bags) + intervention at the small-group level (holding home health party and discussing a specific pesticide topic) +interventions at the individual level (raising knowledge and distributing laundry kits and shower kits)				
			• C: No intervention				
			Measures: Baseline and 2- year follow—up				
Bradman et al. (2009)/ United States of America	To evaluate the efficacy of the intervention in preventing workers and their families pesticide exposure	N = 44	• Farm workers- Multi-faceted interventions:	None specified			Significant difference in level of malathion urinary metabolite between two groups
		I: n = 29					
		C: n = 15	• I: Providing the personal protective equipment + five educational sessions to educate workers				
			• C: Providing personal protective equipment and a short educational course after post test				
			Measures: unclear				
McCauley et al. (2013)/ United States of America	To examine the effects of training intervention on farmworkers participants’ health beliefs, pesticide knowledge, and biomarkers of pesticide exposure	N = 140	• farm workers- Educational/ behavioral Intervention approach:	None specified	Significant difference in knowledge between two gropes (P<0.0001)	Non -significant difference in practice between two groups	Significant differences in two of five urinary pesticide metabolites between two groups
		I: n = 83					
		C: n = 57					
			• I: Providing an education session, a booklet, and a CD containing socio dramas+ contacting by the promotoras to answer questions or providing supplementary explanations				
			• C: an EPA video and a standard EPA brochure.				
			• Measures: Baseline and 6-Week follow- up				
Arcury et al. (2009)/ United States of America	To evaluate the effectiveness of a program on pesticide safety behaviors.	N = 115	• Farm workers and their families- Educational/ behavioral	Theory of reasoned action	Non -significant difference in knowledge between two groups (P>0.05)	Non- significant difference in behaviors between two groups	
		I: n = 65					
		C: n = 50	Intervention approach:				
			• I: Delivering a multi lesson residential pesticide by the promotoras using a variety of media				
			• C: A promotora delivered multi lesson nutrition curriculum				
			• Measures: Unclear				
Salvatore et al. (2009)/ United States of America	To evaluate effects of a community based participatory research worksite intervention on farmworkers’ behaviors	N = 130	• Farmworkers- Multi-faceted interventions:	None specified		Significant differences in four behaviors of 22 assessed behaviors between two groups	
		I: n = 74					
		C: n = 56	• I: Providing four weekly field-based educational sessions + providing hand-washing facilities and personal protective equipment				
			• C: Providing equipment and + education after post- test				
			Measures: Baseline and 2- month follow-up				
Salvatore et al. (2015)/ United States of America	To evaluate the effectiveness of a home-based intervention to reduce pesticide exposures to farmworkers’ children	N = 116	• Farm workers and their families	Health belief model and social cognitive theory			• Non- significant difference in Child urinary metabolites levels between two groups
		I: n = 61	• Educational/ behavioral				
		C: n = 55	Intervention approach:				
			• I: Providing three home- based educational sessions delivered by community health workers				
							• Non- significant difference in pesticide floor wipe except one (Trans—Permethrin, (P<0.05)
			• C: providing a 1-day educational session after post test				
			• Measures: Baseline and 3- month follow up				
Varma et al. (2016)/ Nepal	To evaluate the effectiveness of locally adapted personal protective equipment to reduce organophosphate exposure	N = 90	• Farmers	None specified			Non significant difference in plasma cholinesterase or PChE between two groups
		I: n = 45	• Incentive interventions:				
		C: n = 45	• I: providing locally adapted personal protective equipment				
			• C: using daily practice clothing				
			• Measures: Baseline and Immediately after intervention				
Rattanaselanon et al. (2018)/ Thailand	To evaluate the effectiveness of an occupational health education program to improve the safe handling of chlorpyrifos in farmers	N = 70	• Farmers	Health belief model		Significant difference in safety behaviors between two groups	Significant difference in amount of urinary metabolite
		I: n = 35	• Educational/ behavioral				
		C: n = 35	Intervention approach:				
			• I: Assessing risks individually, providing information, and group discussion				
			• C: No intervention				
			• Measures: Baseline, 2- week, and 4- week follow up				

Note: RCT = randomized controlled trial, N = number, I = intervention group, C = control group or comparison group.

* Knowledge, attitude and beliefs = Knowledge, attitude, beliefs, perceived susceptibility, perceived severity, perceived benefits, perceived barriers, cues to action, safety risk perception.

** Practice or behavior = Practice, use of protective equipment, pesticide storage practices, pesticide safety, safety behavior.

*** Exposure monitoring methods = Hands and urinary metabolites levels, cholinesterase levels, floor wipe levels.

**Table 2:** Effectiveness of controlled pretest/post-test designs to reduce pesticide exposures.

Authors/ Country	Objective of the study	Participants	Intervention focus/	Theoretical framework	Outcomes		
			Intervention approach and strategies/ measures		Knowledge, attitude and beliefs*	Practice or behavior**	Exposure monitoring methods***
Boonyakawee et al. (2013)/ Thailand	To evaluate the effectiveness of intervention to reduce insecticide exposure	N = 92	• Farmworkers	Social cognitive theory	• Significant difference in knowledge between two groups in both follow—ups (P<0.001)		
		I: n = 42	• Educational/ behavioral intervention approach:				
		C: n = 50	• I providing 4-day theoretical and practical training		• Significant difference in attitude between two groups in both follow—ups (P<0.001)		
			• C: No intervention				
			• Measures:				
			Baseline, 2-month, and 5- month follow- up				
Raksanam et al. (2012)/ Thailand	To evaluate the effectiveness of an intervention to improve farmers’ safety behaviors	N = 101	• Farmers	Health belief model	• Significant difference in knowledge between two groups(P<0.001)	• Significant differences in home pesticide safety assessment between two groups (P<0.001)	
		I: n = 50	• Educational/ behavioral intervention approach:				
		C: n = 51	• I: Holding six monthly meetings to discuss on improving hygiene practices+two home visits		• Significant difference in belief between two groups(P<0.001)	• Significant difference in safety behavior between two groups (P<0.001)	
			• C: No intervention				
			• Measures: Baseline and 6- month follow- up				
Suratman et al. (2016)/ Australia	To evaluate the effectiveness of an educational intervention to improve knowledge and perceptions for reducing pesticide exposure	N = 37	• Farmworkers	Health belief model	• Significant difference in knowledge (P<0.001) between two groups		
		I: n = 30	• Educational/ behavioral intervention approach:				
		C: n = 7	• I:providing one group educational session using a PowerPoint presentation		• Significant difference in perceived susceptibility (P<0.001), perceived severity(P = 0.002, perceived barriers(P<0.001), perceived benefit(P = 0.02) between two groups		
			• C: providing one individually educational session using a flipchart				
			• Measures: Baseline and 3- month follow- up Three months after the				
Jors et al. (2014)/ Bolivia	To evaluate the effectiveness of training on pesticide handling and ecological alternatives	N = 70	• Farmers	None specified	• Significant difference in knowledge between two groups in both follow- up	Significant difference in personal protection behavior between two groups in both follow- up	
		I: n = 23	• Educational/ behavioral intervention approach:				
		C: n = 47	• I: Holding 14 theoretical and practical courses for FFS farmers		• Significant difference in attitude between two groups in both follow- up		
			• C: providing two educational courses by FFS farmers to their neighbor farmers				
			• Measures: baseline, 2- year follow- up, 5- year follow- up				
Vela Acosta et al. (2005)/ United States of America	To evaluate _a_ p_esticide_ r_isk_ r_eduction_ p_rogram_	N = 152	• Farm workers- Educational/ behavioral intervention approach:	None specified	• Significant difference in knowledge between two groups (P<0.0001)- Significant difference in safety risk perception between two groups (P<0.0001)	Significant difference in two of safety behavior (out of four) (P> 0.05))	
		I: n = 77	• I: providing one pesticides educational program				
		C: n = 75	• C: Providing intervention after posttest				
			• Measures: Baseline and 1- week follow- up				
Hruska and Corriols. (2002)/ Nicaragua	To evaluate the effectiveness of training in integrated pest management	N = 1260	• Farmers	None specified		Significant difference in insecticide applications between three groups (P<0.0001)	Significant difference in average dose of Methamidophos (P<0.001) and chlorpyriphos (P = 0.017) between three groups
		I_1_: n = 60	• Educational/ behavioral intervention approach				
		I_2_: n = 1200					
		C: n = unknown	• I 1: Providing an intensive training for promoter				
			• I 2: Providing training by promoters				
			• C: No intervention				
			• Measures: Baseline and two-year follow- up				
Vaidya et al. (2017)/Nepal	To evaluate the effectiveness of a training program to increase awareness on harmful effects of pesticides and to enhance capacity for safe	N = 249	• Farmers	None specified	• Significant difference in knowledge between three groups (P<0.05)	Significant difference in practice between three groups (P<0.05)	
		I_1_: n = 57	• Educational/ behavioral intervention approach:				
		I_2_: n = 98					
		C: n = 94	• I1: Providing 10 to 15 theoretical and practical courses about IPM, health, and environment for key farmers		• Significant difference in attitude between three groups (P<0.05)		
			• I2: providing training for farmers by key farmers				
			• C: No intervention				
			• Measures: baseline and unclear follow—up				

Note: cPPI = controlled pretest/post-test interventions, PPI = pretest/post-test interventions, N = number, I = intervention group, C = control group or comparison group.

* Knowledge, attitude and beliefs = Knowledge, attitude, beliefs, perceived susceptibility, perceived severity, perceived benefits, perceived barriers, cues to action, safety risk perception.

** Practice or behavior = Practice, use of protective equipment, pesticide storage practices, pesticide safety, safety behavior.

*** Exposure monitoring methods = Hands and urinary metabolites levels, cholinesterase levels, floor wipe levels.

**Table 3:** Effectiveness of pretest/ post-test designs to reduce pesticide exposures.

Authors/ Country	Objective of the study	Participants	Intervention focus/	Theoretical framework usage and	Outcomes		
			Intervention approach and strategies/ measures		Knowledge, attitude and beliefs*	Practice or behavior**	Exposure monitoring methods***
LePrevost et al. (2014)/ United States of America	To evaluate the effectiveness of a pesticide and safety curriculum	N = 20	• Farm workers	None specified	Significant change in knowledge (P< 0.001)		
		I: n = 20	• Educational/ behavioral intervention approach:				
			• I: Providing Pesticides and Farmworker Health Toolkit consisted of training flip chart with a trainer’s guide and visual materials for the audience; hands-on learning activities; and a one-page, take-home handout				
			• Measures: Baseline and unclear follow- up				
Elkind et al. (2002)/ United States of America	To evaluate the effectiveness of theater to improve farm health and safety knowledge	N = 301	• Farm workers and their families	Observational learning theory	Significant change in knowledge(P<0.010)		
		I: n = 185 and 115					
			• Educational/ behavioral				
			Intervention approach:				
			• I: Providing educational theater				
			• Measures: Baseline and 2- month follow- up				
Napolitano et al. (2002)/ United States of America	To develop and evaluate a pesticide education video	N = 41	• Farm workers and their families	None specified	Significant change in knowledge (P<0.0001)	• Non-significant change in behaviors at work	
		I: n = 27 and 14	• Educational/ behavioral intervention approach:				
			• I: Providing training using a developed educational video			• Significant change in behaviors at home (P = 0.023)	
			• Measures: Baseline and 1- week follow- up				
Janhong et al. (2005)/ Thailand	To evaluate the effectiveness of health promotion program for the safe use of pesticides	N = 33	• Farmers	None specified	• Significant change in knowledge (P<0.000)	Significant change in practice (P<0.000)	
		I: n = 33	• Educational/ behavioral intervention approach:				
			• I Providing educational sessions and sending appointments reminder cards		• Significant change in attitude (P<0.000)		
			Measures: Baseline and 6- month follow-up				
Sam et al. (2008)/ India	To evaluate the effectiveness of educational program to promote pesticide safety	N = 76	• Farmers and their families	None specified	• Significant change in knowledge only from baseline to the first follow-up (P<0.001)	Significant change in practice from baseline to the second follow- up	
		I: n = 76	• Educational/ behavioral intervention approach:		• Significant change in attitude from baseline to the first and second follow- up (P<0.001)		
			• I: Providing a 15–30 min educational session				
			• Measures: Baseline, immediately, and one month after intervention				
Orozco et al. (2011)/ Ecuador	To evaluate the effectiveness of a community-based program to changes in health promotion outcomes relevant to highly hazardous pesticide use	N = 359	• Farmers and their families	None specified	• Significant change in household managers’ knowledge P< 0.05).	• Significant change in household managers’ practice P< 0.05).	
		I: n = 359	• Multi-faceted interventions:				
			• I: Community interventions (including radio spots and monthly educational sessions delivered in schools, community centers, and farmers’ fields)+agricultural interventions (holding agricultural workshops and rotating funds to purchase protective equipment)				
			• Measures: Baseline and 6-minth follow-up				
Liebman et al. (2007)/ United States of America	To evaluate the effectiveness of an educational interventions about the risks from pesticide exposure	N = 273	• Farm workers and their families	None specified	Significant change in knowledge (P<0.05)	Significant change in behavior (P<0.05)	
		I: n = 273					
			• Educational/ behavioral intervention approach:				
			• I: Training farmworkers and their families during home visits and small group workshops by promotoras				
			• Measures: Baseline and 4-week follow—up				
Weerasinge et al. (2008)/Sri Lanka	To identify the important design features influencing community acceptance and use of safe storage devices	N = 368 (200 from phase 1 and 168 from phase 2)	• Farmers and their families	None specified		• Significant change in the number of households storing pesticides in lock storage devices (P<0.05)- Significant change in storage of pesticides in the field from (P<0.05)	
			• Multi-faceted interventions:				
		I: n = 368	• I: Providing safe storage devices+ holding home visits to provide education about how to use safe storage boxes box				
			• Measures: Baseline, 7- month and 24- month follow up				
Konradsen et al. (2007)/ Sri Lanka	To evaluate the effectiveness of an intervention to change community perceptions and use of in-house safe storage boxes for pesticides	N = 172	• Farmers and their families	None specified		• Significant change in number of households keeping pesticide safe from children (P<0.001) and adult (P<0.05)Significant reduce in the number of households storing pesticide in the field (P<0.05)	
		I: n = 172	• Multi-faceted interventions:				
			• I: Providing education on the use of the box+ home visit + providing safe storage boxes			• Significant reduce in the number of households keeping pesticide openly in house (P<0.001)- Significant increase in the number of households keeping pesticide in house under lock (P<0.001)	
			• Measures: Baseline and 7- month follow- up			• Significant reduce in the number of households keeping pesticide in house under lock (P<0.001)	
Cole et al. (2007)/ Ecuador	To evaluate the effectiveness of community-based interventions to reduce the pesticide exposures and associated neurotoxic burden	N = 138	• Farmers and their families	None specified	Significant change in pesticide-related knowledge (P < 0.004)	• Significant change in general pesticide practices (P = 0.001)	Significant change in neurobehavioral tests (P<0.0001)
		I: n = 138	• Multi-faceted interventions: I: Community awareness-raising activities (such as community fairs and radio spots)+household level education (such as home visit and group discussions on priority concerns) + providing no-interest credit towards the purchase price of PPE			• Significant change in use of PPE (P<0.001)	
			• Measures: Baseline and 1- year follow-up				
Snipes et al. (2015)/ United States of America	To evaluate the effectiveness of a mobile platform to promote PPE usage	N = 55	• Farm workers	None specified		Significant change in two out of five personal protective equipment use (P<0.01)	
		I: n = 55	• Multi-faceted interventions:				
			• I: Providing the gloves, glasses and long sleeved shirts+ sending tailored risk reduction messages through the app				
			• Measures: Baseline and unclear follow- up				
Nazari and Bin Hj Hassan. (2011)/ Iran	To evaluate the effectiveness of using television as an educational tool for the enhancement of farmers’ knowledge	N = 161	• Farmers	None specified	Significant improvement in knowledge (P<0.001)		
		I: n = 161	• Educational/ behavioral intervention approach:				
			• I: creating and providing an educational film by the local television center				
			• Measures: baseline and 1- week follow-up				
Anger et al. (2009)/ United States of America	To evaluate the effectiveness of training for improve the knowledge about pesticide exposure	N = 61	• Farm workers	None specified	Significant improvement in knowledge (P<0.001)		
		I: n = 61	• Educational/ behavioral intervention approach				
			• I: providing a computer- based training				
			• Measures: Baseline and 5-month follow-up				
Quandt et al. (2013)/ United States of America	To evaluate the effectiveness of intervention to change in knowledge of pesticide dangers and practices related to residential pesticide exposure	N = 610	• Farmers and their families- Educational/ behavioral intervention approach:	Health belief model	Significant improvement in knowledge (P<0.001)	Significant improvement in practices (P<0.001)	
		I: n = 610	• I: Providing one-on-one education by promoters through home visits				
			• Measures: Baseline and 18- month follow-up				
Zalat et al. (2015)/ Egypt	To evaluate the effectiveness of an intervention to change in knowledge, attitude, and behaviors toward pesticides exposure	N = 202	• Farmers and their families	Health belief model	• Significant improvement in knowledge (P<0.05)	Non- significant change in behaviors	
			• Multi-faceted interventions:				
			• I: Providing training by holding small group discussions and lectures+ offering gloves				
		I: n = 202	• Measures: Baseline and 3-month follow- up		• Non- significant change in attitude		

Note: cPPI = controlled pretest/post-test interventions, PPI = pretest/post-test interventions, N = number, I = intervention group, C = control group or comparison group.

* Knowledge, attitude and beliefs = Knowledge, attitude, beliefs, perceived susceptibility, perceived severity, perceived benefits, perceived barriers, cues to action, safety risk perception.

** Practice or behavior = Practice, use of protective equipment, pesticide storage practices, pesticide safety, safety behavior.

*** Exposure monitoring methods = Hands and urinary metabolites levels, cholinesterase levels, floor wipe levels.

### Quality assessment

The quality of included studies was assessed by two independent reviewers (M.A and S.K) using the Effective Public Health Practice Project quality assessment tool (EPHPP). This tool is applicable across multiple study designs. EPHPP rates studies as strong, moderate, and weak in terms of their data collection methods, confounders, study design, selection bias, blinding, dropouts, and intervention integrity [15]. Any discrepancy between two reviewers on the quality rating process was resolved by discussion or by a third reviewer (A. K-Sh).

Inter-rater reliability was approved by calculating the percentage of conformity and a Cohen’s Kappa coefficient [16]. We did not exclude studies based on the results of quality assessment. Inter-rater agreement varied across EPHPP component ratings. Overall, there was a good agreement between the two reviewers (Kappa coefficient ranged from 0.66 to 1.00).

## Results

### Results of the search and included studies

**Fig 1:**
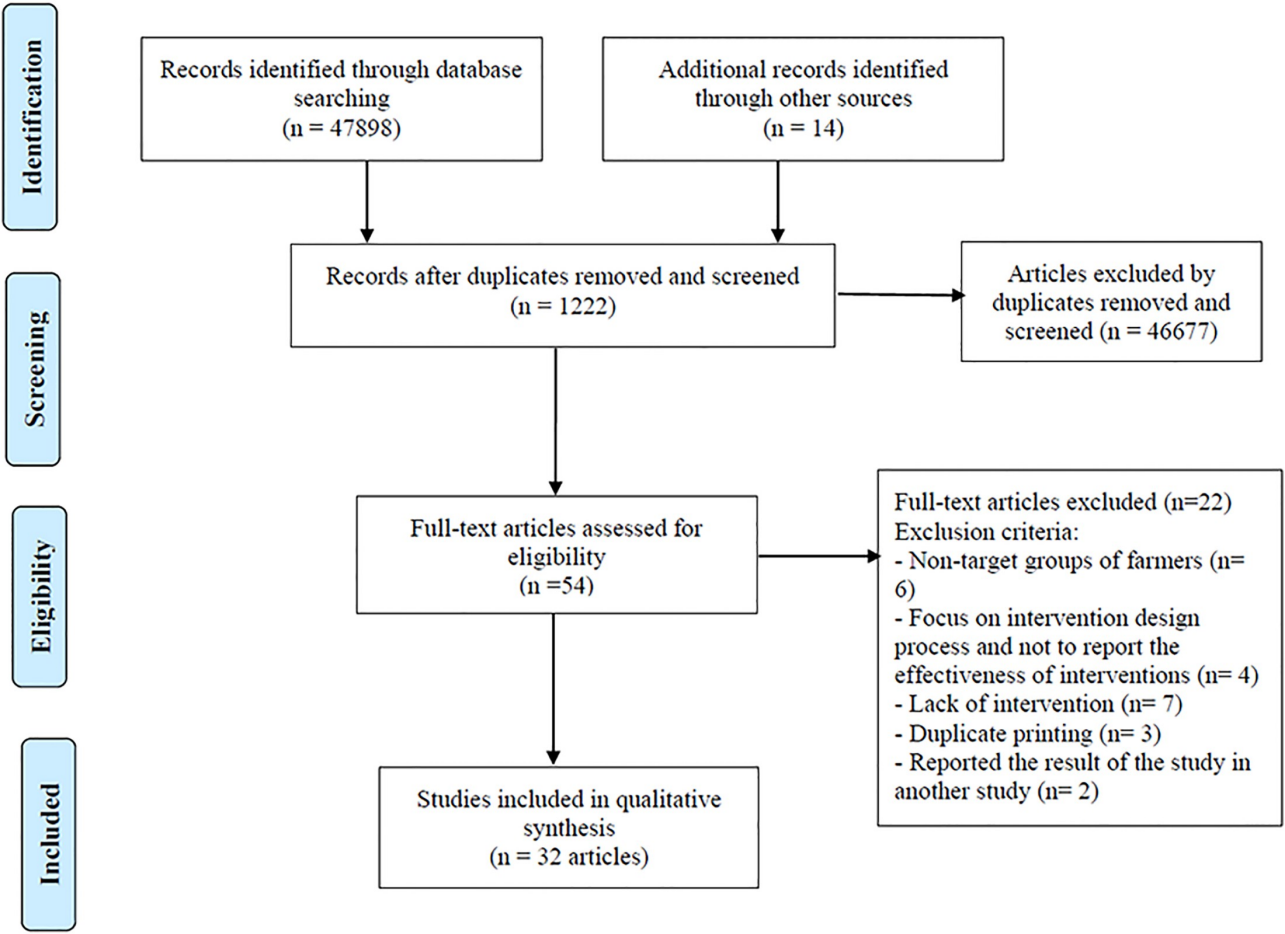
Flow diagram for the identification, screening, eligibility and inclusion of studies.

A total of 47912 references were identified and reviewed. From these references: 1222 were selected for abstract review and after in-depth abstract review, and 54 were selected for detailed review. Then reference lists of these articles were checked and 14 new references were identified. Finally, we included 32 articles from 31 studies in this review that satisfied our inclusion criteria (Fig 1). A summary of the included articles is provided in Tables 1, 2 and 3. Results of the Quandt et al study [17] were reported in the article by Grzywacz et al [18]. Therefore, Quandt et al [17] was considered as the main study. The results of Farahat et al study (2008) [19] were reported in Farahat et al study [20] and Farahat et al study was considered as the main study [20]. Thus, two articles came out. Thompson et al. [21] and Strong et al [22] reported the results of a study in the form of two articles (Fig 1).

### Design of the studies

Of the 31 studies, seven studies were controlled pretest/post-test (cPPI) interventions [5, 23–28], fifteen studies were pretest/post-test interventions (PPI) [17, 29–42], and nine studies were randomized controlled trials (RCT) [20, 22, 43–49].

### Study time and settings

Fifteen articles were published in 2010 or later [5, 17, 23, 24, 26, 28, 34, 37, 38, 40, 42, 45, 47–49]. Fourteen studies were carried out in the USA [17, 22, 27, 29, 31, 34–36, 40, 43–47]. The other eighteen studies were conducted outside the USA: four in Thailand [23, 24, 32, 49], two in Sri Lanka [33, 41], two in Ecuador [30, 38], two in Egypt [20, 42], two in Nepal [28, 48] and one in India [39], Iran [37], Australia [26], Bolivia [5], and Nicaragua [25].

### Participants and follow-up duration

Most studies had a small sample size. The number of participants in twenty-one studies was less than 200 [5, 23, 24, 26, 27, 29, 30, 32–34, 36, 37, 39, 40, 43–49]. Sixteen studies focused on farmers/ farmers and their families [5, 17, 20, 24, 25, 28, 30, 32, 33, 37–39, 41, 42, 48, 49]. Sixteen studies were conducted among farmworkers/ farmworkers and their families [21–23, 26, 27, 29, 31, 34–36, 40, 43–47]. The follow-up duration for studies was often quite short, one month or less in eight studies [20, 27, 35–37, 39, 48, 49], less than 6 months in eight studies [23, 26, 29, 31, 42, 45–47], and more than 6 months in ten studies [5, 17, 22, 24, 25, 30, 32, 33, 38, 41]. Follow-up in five studies was unknown [28, 34, 40, 43, 44].

### Theoretical framework usage

Of included studies, only eight (25%) used the behavioral theories. The health belief model (HBM) was the most frequent theoretical framework employed [17, 24, 26, 42, 47, 49]. Other theories included social cognitive theory [23], observational learning theory [31], and the theory of reasoned action [43]. One study applied both the health belief model and social cognitive theory [47]. Of these studies, only in two studies, components of theories were measured [24, 27] and theoretical frameworks in the remaining studies were used only to guide the intervention development [17, 23, 31, 42, 43, 47, 49].

### Types of outcome measures

Results of the included articles were mainly based on self-reported data and only in eight articles objective measures (such as levels of urinary malathion metabolites, dislodgeable foliar residue (DFR), and plasma cholinesterase) were used to evaluate the effects of interventions [21, 25, 30, 44, 45, 47–49]. Among these articles, five used both objective and self-reported [25, 30, 45, 48, 49]. Among the articles relying on self-reported data, seven articles targeted at knowledge and attitude/beliefs of participants [23, 26, 29, 31, 34, 37, 42], twelve articles tested knowledge, attitude/ beliefs as well as behavior/ performance of the participants [5, 17, 20, 24, 27, 28, 32, 35, 36, 38, 39, 43], and five articles assessed only the behavior/performance of participants [22, 33, 40, 41, 46].

### Types of intervention and their effects

Due to the heterogeneity designs and outcome variables of included studies, qualitative and semi-quantitative analyses were used to analyze the results.

In order to categorize the interventions, we used the categories provided by Murphy (1980) and Haddon (1996) which divided interventions into five groups, including education/ behavior change, incentive, engineering/ technology, legislation/ enforcement, or multifaceted programs [10].

#### Educational/behavioral intervention

Educational/ behavioral approach (e.g. training the farmers and their families at workplace or through home visit) was used in twenty-one studies [5, 17, 20, 23–29, 31, 32, 34–37, 39, 43, 45, 47, 49]. Five of these studies were RCTs [20, 43, 45, 47, 49], whereas seven were controlled in pretest/post-test interventions (cPPI) [5, 23–28], and nine employed pretest/post-test designs without control group (PPI) [17, 29, 31, 32, 34–37, 39]. Ten of these studies focused on farmers/farmers and their families [5, 17, 20, 24, 25, 28, 32, 37, 39, 49], and eleven studies were conducted among farmworkers/farmworkers and their families [23, 26, 27, 29, 31, 34–36, 43, 45, 47], (Tables 1, 2, and 3). Of the 21 studies that applied educational/ behavioural approach, 14 studies (67%) were assessed as having low quality [5, 20, 24, 25, 27–32, 34, 36, 43, 49] (Table 4).

**Table 4:** Quality assessment using EPHPP quality rating.

Authors/ Country	Selection bias	Study design	Blinding	Confounders	Data collection methods	Withdrawals and drop-outs	Study quality
Farahat et al. (2009)/ Egypt	Moderate	Strong	Moderate	Weak	Strong	Weak	Weak
Thompson et al. (2008)/ United States of America	Moderate	Strong	Moderate	Weak	Weak	Strong	Weak
Strong et al. (2009)/ United States of America	Moderate	Strong	Moderate	Weak	Weak	Strong	Weak
Bradman et al. (2009)/ United States of America	Moderate	Strong	Moderate	Weak	Weak	Weak	Weak
McCauley et al. (2013)/ United States of America	Moderate	Strong	Moderate	Strong	Weak	Strong	Moderate
Arcury et al. (2009)/ United States of America	Moderate	Strong	Moderate	Strong	Weak	Weak	Weak
Salvatore et al. (2009)/ United States of America	Moderate	Strong	Moderate	Weak	Strong	Moderate	Moderate
Salvatore et al. (2015)/ United States of America	Moderate	Strong	Moderate	Strong	Moderate	Strong	Strong
Varma et al. (2016)/ Nepal	Moderate	Moderate	Moderate	Weak	Moderate	Weak	Weak
Rattanaselanon et al. (2018)/ Thailand	Moderate	Strong	Moderate	Weak	Weak	Weak	Weak
Boonyakawee et al. (2013)/ Thailand	Moderate	Moderate	Moderate	Strong	Strong	Weak	Moderate
Raksanam et al. (2012)/ Thailand	Weak	Moderate	Moderate	Strong	Strong	Weak	Weak
Suratman et al. (2016)/ Australia	Moderate	Moderate	Moderate	Strong	Strong	Weak	Moderate
Jors et al. (2014)/ Bolivia	Moderate	Moderate	Moderate	Strong	Weak	Weak	Weak
Vela Acosta et al. (2005)/ United States of America	Moderate	Moderate	Moderate	Weak	Weak	Strong	Weak
Hruska and Corriols. (2002)/ Nicaragua	Moderate	Moderate	Moderate	Weak	Strong	Weak	Weak
Vaidya et al. (2017)/ Nepal	Moderate	Moderate	Moderate	Strong	Weak	Weak	Weak
LePrevost et al. (2014)/ United States of America	Moderate	Moderate	Moderate	Weak	Strong	Weak	Weak
Elkind et al. (2002)/ United States of America	Moderate	Moderate	Moderate	Weak	Moderate	Weak	Weak
Napolitano et al. (2002)/ United States of America	Moderate	Moderate	Moderate	Weak	Weak	Weak	Weak
Janhong et al. (2005)/ Thailand	Moderate	Moderate	Moderate	Weak	Strong	Weak	Weak
Sam et al. (2008)/ India	Moderate	Moderate	Moderate	Weak	Moderate	Strong	Moderate
Orozco et al. (2011)/ Ecuador	Moderate	Moderate	Moderate	Weak	Weak	Weak	Weak
Liebman et al. (2007)/ United States of America	Strong	Moderate	Moderate	Weak	Moderate	Strong	Moderate
Weerasinge et al. (2008)/	Moderate	Moderate	Moderate	Weak	Weak	Strong	Weak
Sri Lanka							
Konradsen et al. (2007)/	Moderate	Moderate	Moderate	Weak	Weak	Strong	Weak
Sri Lanka							
Cole et al. (2007)/ Ecuador	Strong	Moderate	Moderate	Strong	Weak	Weak	Weak
Snipes et al. (2015)/ United States of America	Moderate	Moderate	Moderate	Weak	Weak	Moderate	Weak
Nazari and Bin Hj Hassan. (2011)/ Iran	Moderate	Moderate	Moderate	Weak	Strong	Strong	Moderate
Anger et al. (2009)/ United States of America	Moderate	Moderate	Moderate	Weak	Weak	Strong	Weak
Quandt et al. (2013)/ United States of America	Moderate	Moderate	Moderate	Weak	Strong	Strong	Moderate
Zalat et al. (2015)/ Egypt	Moderate	Moderate	Moderate	Weak	Moderate	Weak	Weak

Study quality = 1: Strong (no weak ratings); 2: Moderate (one weak rating); 3: Weak (two or more weak ratings).

Eighteen studies (including 8 studies among farmers/farmers and their families and 10 studies among farmworkers/ farmworkers and their families) categorized in the educational/ behavioral approach measured the participants’ knowledge as an outcome [5, 17, 20, 23, 24, 26–29, 31, 32, 34–37, 39, 43, 45]. The result of these studies showed that all but one study conducted among farm workers and their families were successful [43]; however, the quality of about 59% of these interventions was graded low [5, 20, 24, 27, 28, 29, 31, 32, 34, 36].

All seven studies (including four studies among farmers/farmers and their families and 3 studies among farmworkers/ farmworkers and their families) which measured the participants’ attitude/ beliefs [23, 24, 26–28, 32, 39] were effective. However, four studies were assessed to have low quality [24, 27, 28, 32].

Participants’ behaviour /practices were considered as outcome in fourteen studies (including 9 studies among farmers/farmers and their families and 5 studies among farmworkers/ farmworkers and their families), participants’ behaviour /practices were considered as outcome variable [5, 17, 20, 24, 25, 27–28, 32, 35, 36, 39, 43, 45, 49]. Of these studies three were not successful [20, 43, 45], and two improved in some assessed behaviour and practices [27, 36]. The quality of about 45% of effective interventions was graded low [5, 24, 25, 28, 32, 49]. Interestingly, while about 89% of studies targeted farmers/ farmers and their families were successful in making a significant change in behavior/ practice [5, 17, 24, 25, 28, 32, 39, 49], only 20% of studies targeted farmworkers/ farmworkers and their families have been reported as completely effective.

Of educational/ behavioral interventions, only four studies (including two studies among farmers and two studies among farmworkers/ farmworkers and their families) evaluated the effectiveness using objective measures (e.g., biomarkers of pesticide exposure and neurobehavioral test) [25, 45, 47, 49]. While of four studies, two conducted among farmers were found effective [25, 49], none of the two studies conducted among workers were completely effective [45, 47].

#### Incentive intervention

Incentive intervention consisted of providing money/PPE or positive feedback. We found only one study that specifically evaluated an incentive intervention [48]. The results revealed that it was not effective in making a change in acute organophosphate poisoning symptoms and plasma cholinesterase.

#### Engineering and technological interventions

Engineering/technology interventions consisted of improving the pesticide sprayer machines and equipment and replacing hazardous structures with safer ones. We did not find any studies that specifically evaluated only an engineering and technological intervention.

#### Legislation/enforcement intervention

We did not find any studies that specifically evaluated only a legislation/enforcement intervention.

#### Multifaceted programs

Multifaceted programs were applied in ten studies (including 5 studies among farmers/farmers and their families and 5 studies among farmworkers/ farmworkers and their families) [21, 22, 30, 33, 38, 40–42, 44, 46] which included a combination of interventions such as education, home visits, providing personal protective equipment (e.g., coveralls and gloves), laundry service, warm water and soaps, containers for storing work shoes and clothes. Of these studies, four were RCT [21, 22, 44, 46], and six employed quasi-experimental designs without control group (PPI) [30, 33, 38, 40–42]. Of ten multifaceted programs, nine studies were assessed to have a low-quality [21, 22, 30, 33, 38, 40–42, 44] (Table 4).

Of nine studies that used a multifaceted approach, three low-quality studies assessed the participants’ knowledge as an outcome, which was effective [30, 38, 42]. In eight studies, participants’ behavior/ practices were considered as the outcome [22, 30, 33, 38, 40–42, 46]. Of these studies four low-quality studies which focused on farmers/ farmers and their families were successful in making change in participants ‘behaviors [30, 33, 38, 41], whereas three studies which focused on farmworkers/ farmworkers and their families improved in some assessed behavior and practices [22, 40, 46]. In three studies, objective measures were used to evaluate the effectiveness of the multifaceted programs [21, 30, 44]: two among farmworkers and one among farmers. Of these studies two were effective in assessed metabolites [30, 44].

## Discussion

To the best of our knowledge, there are no systematic reviews that examine comprehensively the effectiveness of interventions to promote pesticide safety and reduce pesticide exposure among farmers or farmworkers. Indeed, we conducted this review to address the gaps of previous efforts. The majority of the studies included were quasi-experimental (21/31). This might be due to the affordability and feasibility of this type of design. Moreover, 75% (24/32) of articles were categorized as low quality.

Although we expected to find evidence on the effectiveness of five interventional approaches, we discovered no studies investigating specifically the legislation/ enforcement or engineering/ technology interventions. We also found only one study that specifically addresses the effectiveness of the incentive intervention. However, this type of intervention was used in combination with other types of interventions (i.e., multifaceted intervention). The majority of studies were based specifically on educational/ behavioral interventions. In addition to studies that specifically evaluated educational interventions, this approach has been used in all the multifaceted interventions as a key component. Although the evaluated educational interventions were effective in general, the following two considerations should be taken into account in the interpretation of results: First, the main success of these studies was to change participants’ knowledge and beliefs and in cases that practices and objective measures were considered as the outcomes, the interventions were less likely to be successful. Second, the positive results of these interventions should be interpreted in light of the design and quality of studies.

The majority (68.8%) of the studies used this approach were quasi-experimental designs and about 71% were categorized as low-quality studies and only one study was ranked as having a strong quality. This case becomes clear considering that in terms of all outcomes; results of quasi-experimental studies were more successful than the findings of RCTs. This could be related to the inherent weakness of quasi-experimental design for evaluating the effectiveness of alternative intervention rigorously. Similar concerns have been raised about other reviews evaluating the effectiveness of educational interventions among farmers and farmworkers [12, 50].

Another finding of our review is that compared to farmworkers and their families, the interventions which targeted farmers and their families achieved more success. There are some explanations for this result. Farmworkers may have lower-incomes, lower education levels, cultural /language barriers, and in some cases temporary work permits. Also, they may have less control over the work conditions [51].

This review highlights the limited research on this field in LMICs. While only 3.15% of total employment in HICs (vs. 36.15 in low-middle income countries) relates to the agriculture sector [1] more than 50% of the identified studies were based on data from HICs. Also, regarding the type of design of the included studies, our review showed that seven out of nine studies which used RCT were conducted in USA which highlights the challenges associated with performing RCT in LMICs.

The multi-faceted intervention would be expected to reveal more positive results than educational/ behavioral interventions. However, our review showed that this type of intervention was partially successful in changing promote pesticide safety and reduce pesticide exposure It should be noted that multifaceted interventions in the included studies consisted of a combination of educational/ behavioral and incentive interventions. In other words, no multi-faceted intervention studies used engineering/ technology or legislation/ enforcement approach as part of their intervention strategy. This might be due to the implementation challenges and limited infrastructure. It seems that to be effective, educational/ behavioral interventions need to be embedded in contextual factors and combined with engineering/ technology or legislation/enforcement approaches. Although there are many laws and regulations to protect farmers and farmworkers from pesticide risks-especially in HICs- investigation on their adaptation, implementation, and evaluation remain underexplored in the literature.

Regardless of study design and type of intervention used, our review showed that the interventions were most likely to be successful in making a change in participants’ knowledge/ beliefs. However, it should be noted that only in seven studies (out of 21) that evaluated knowledge/ beliefs, follow up duration was longer than six months. This means that it is not clear that the obtained changes would be sustained over time.

Only eight articles (out of 32) determined objective measures to evaluate the effects of interventions. This may be due to several reasons: lack of skill in evaluation plan, insufficient funding, and supervision, and lack of available resources and equipment [52].

Regarding outcomes other than knowledge/ beliefs, the success of interventions was partial. We believe that there are at least four explanations for this finding. First, while the increasing evidence suggests that the legislation/enforcement approach had an influence on the effectiveness and sustainability of health-related interventions in other contexts [53, 54], the findings of this review showed that the majority of studies used only educational/ behavioral approaches.

Health promotion activities can play a significant role in increasing visibility and highlighting the importance of legislation/ enforcement interventions. Given the fact that the effects of the legislation/enforcement or engineering/ technology interventions do not appear immediately, design studies such as time series may be used to evaluate these types of interventions. Second, this may reflect lack of attention to behavior change technique (BCT) according to targeted outcomes. Indeed, it seems that the majority of included studies involved providing the overall information regarding the risks and behavior and did not apply behavioral methods such as goal setting, planning/ implementation, and social encouragement/ support. There is evidence suggesting that providing individually tailored information (vs. overall information [55] and using particular behavior change techniques are associated with more effectiveness [56]. We recommend a review for future focusing on comparisons of the different behavior change methods. Third, it is evident that considering social and behavioral science theories in developing public and health promotion programs is associated with more effectiveness [10]. These frameworks help to understand health behaviors and the contexts (such as cultural, economic, and social circumstances) in which they occur. Our findings revealed that a majority of studies (about 71%) did not explicitly apply social or behavioral theories. Fourth, it should be taken into account that changing the behavior of farmers and farmworkers is difficult and many protective recommendations are never adopted by farmers. Based on the ecological model, for attempts to be effective in farmers’ and farmworkers’ health and modify farmers/farmworkers’ behavior, it is necessary to develop multilevel interventions (i.e., intervention at individual, interpersonal, organizational, community, and public policy level) targeting different barriers. The results of this study and the existent evidence show that most programs in this area tend to focus on the individual farmers/ farmworkers and limited attention has been paid to involving the higher layer of the ecological model. In this study, we found that the focus of about 54% of the studies was on the farmers/farmerworkers, and the rest of the interventions had some components for their families. In this way, the higher layers of ecological model have been ignored.

This review has limitations. We included multiple types of outcomes and study designs which makes it impossible to perform a meta-analysis. In addition, in order to assess the higher quality evidence of the effectiveness of the intervention, we didn’t search the grey literature; therefore, a publication bias may exist in this review.

## Conclusion

The studies included in this review addressed the effectiveness of interventions for farmers/ farmworkers to promote pesticide safety and reduce pesticide exposure. The majority of studies relied on only education/ behavior change. We also found some studies that reported the effects of multifaceted programs combining more than one approach such as education, home visits, and providing personal protective equipment. Although the interventions were effective in general, the results should be interpreted in the light of design limitations and self-reported outcomes. Further research is crucial to understand the role of other than educational/ behavioral interventions and the effectiveness of well-designed on more objective outcomes.

## Supporting information

S1 Appendix Search strategy for PubMed. (DOCX)

S1 File PRISMA 2009 checklist. (DOC)
