# Supplementary material for: Effectiveness of interventions to promote pesticide safety and reduce pesticide exposure in agricultural health studies: A systematic review
Source: PLoS One. 2021 Jan 26;16(1):e0245766. doi: 10.1371/journal.pone.0245766 (PMC7837484; doi:10.1371/journal.pone.0245766)
Supplement: S1 Appendix — (DOCX) [file pone.0245766.s001.docx]

**S1 Appendix.**

Search strategy for PubMed

Agricultur* OR agriculture OR farm OR farms OR farmer* OR farming OR "farm worker" OR "farm workers" OR "farmworker" OR "farmworkers" OR ranch* OR dairy OR dairying OR dairies OR greenhous* OR orchard* OR livestock* OR "live stock" OR "live stocks" OR "animal confinement" OR "farm animal" OR "crop production" OR harvesting OR horticultur* OR horticulture OR agronom* OR mix* OR cattle OR animals OR work OR occupation OR gardening OR "crops, agricultural" OR "agricultural workers' diseases" OR gardener OR "agricultural worker" OR "fruit grower" OR orchardist OR grower OR cultivator OR planter

AND

Pesticid* OR pesticides OR fungicid* OR herbicid* OR organophosphate OR biocides OR herbicide OR insecticid* OR carbamat* OR carbamates OR pyrethrins OR molluscacid* OR rodenticid* OR poison* OR "pesticide exposure" OR fungicide OR fumigant

AND

Intervention OR effect* OR "personal protective equipment" OR "safety behavior" OR safety OR prevention OR glove OR mask OR boots OR control* OR protect OR evaluation* OR program* OR training OR "educational program"

AND

Trial OR "randomized controlled trial" OR "quasi-randomized controlled trials" OR "quasi-randomized controlled trial" OR "cluster-randomized controlled trial" OR "cluster-randomized controlled trials" OR "controlled clinical trial" OR "randomized controlled trials" OR "random allocation" OR "double-blind method" OR "single-blind method" OR "clinical trial" OR "clinical trials" OR singl* OR doubl* OR trebl* OR tripl* OR mask* OR blind* OR "latin square" OR placebos OR placebo* OR random* OR "research design" OR "comparative study" OR "evaluation studies" OR "follow up studies" OR "prospective studies" OR "cross-over studies" OR control* OR prospectiv* OR volunteer* OR human NOT animal
