# Supplementary material for: Effectiveness of interventions to promote pesticide safety and reduce pesticide exposure in agricultural health studies: A systematic review
Source: PLoS One. 2021 Jan 26;16(1):e0245766. doi: 10.1371/journal.pone.0245766 (PMC7837484; doi:10.1371/journal.pone.0245766)
Supplement: S1 File — (DOC) [file pone.0245766.s002.doc]

| **Section/topic** | **#** | **Checklist item** | **Reported on page #** |
| --- | --- | --- | --- |
| **TITLE** | | |  |
| Title | 1 | Identify the report as a systematic review, meta-analysis, or both. | Effectiveness of interventions to promote pesticide safety and reduce pesticide exposure in agricultural health studies: A systematic review  (Page 1, title section) |
| **ABSTRACT** | | |  |
| Structured summary | 2 | Provide a structured summary including, as applicable: background; objectives; data sources; study eligibility criteria, participants, and interventions; study appraisal and synthesis methods; results; limitations; conclusions and implications of key findings; systematic review registration number. | Objective: There is a relationship between pesticide exposure and farmworkers’ health. Well-conducted evaluations can provide an insight into how to develop and implement more effective interventions to prevent farmers and farmworkers' exposure to pesticides. This review aimed to summarize the literature on the effectiveness of interventions to promote pesticide safety and reduce pesticide exposure among farmers and farmworkers.  Methods: A comprehensive search on PubMed, Embase, ISI Web of Science, Scopus, Science Direct, Agricola, NIOSHTIC, and Agris databases was performed to identify relevant studies published from 2000 to 2019. Randomized controlled trials (RCTs) and quasi-experimental studies assessing the effectiveness of interventions on a variety of outcomes related to pesticide exposure were considered. The searches were restricted to articles written in English. The methodological quality of included reviews was appraised using the Effective Public Health Practice Project quality assessment tool (EPHPP).  Results: The initial search led to 47912 records, 31 studies of which including nine RCTs and twenty-two quasi-experimental studies met the criteria. The majority of the included studies focused on the educational/ behavioral approach. The studies that applied this approach were effective in improving the participants’ knowledge and attitude; however, these interventions were less effective in terms of making changes in participants’ behaviors and their risk of exposure to toxic pesticides. Multifaceted interventions were moderately effective in terms of improving farmers' and farmworkers' behaviors and reduction in exposure to toxic pesticides. We did not find any studies that had evaluated the effectiveness of engineering /technological, and legislation/enforcement interventions.  Conclusions: Although the majority of studies were based on an educational/behavioral approach and did not assess the effect of interventions on objective measures, the results of this review highlight the significant effectiveness of educational programs and some potential key elements of these interventions. These findings may inform policymakers to develop interventions to reduce pesticide exposure among farmers and farmworkers.  (Pages 1 and 2 , The Abstract section)  This project has been approved by the Research and Technology Deputy of Hamadan University of Medical Sciences.  (Page 17, the Aknowledgment section) |
| **INTRODUCTION** | | |  |
| Rationale | 3 | Describe the rationale for the review in the context of what is already known. | Agriculture is a pillar of development and achieving the goal of sustainable agriculture is one of the most important goals and responsibilities of each country. As reported by the World Bank (2010), about 20% of workers were employed in agriculture worldwide. Regardless of working conditions, due to exposure to hazardous factors, agriculture has been ranked as one of the most hazardous industries.  The use of pesticides may cause serious health problems in farmers and farmworkers. Pesticide use is dramatically increasing worldwide. Optimum and safe use of pesticides and chemical fertilizers are essential to save agricultural products from poisonous contamination and farmers' health. However, along with the advantages of pesticides, there are also threats such as environmental pollution and acute pesticide poisonings due to improper and unsafe handling.  Several factors associated with farmers' pesticide exposure have been reported in previous investigations. Some of these factors include using banned or restricted pesticides, the lack of use of appropriate equipment along with taking protective activities that are needed during pesticide handling, label non-compliance, improper spraying or protective equipment, unsafe pesticide disposal, insufficient knowledge of the law, limitations of environmental standards, and lack of training.  Existing evidence on farmers and farmworkers' health highlights a research gap in this regard. Particularly, the effectiveness of varied interventions developed to reduce agricultural injuries is not well documented. The well-conducted evaluations would increase our knowledge of the prevention of farmers' pesticide exposure by documenting what works. These efforts could be useful in identifying the most cost-effective programs in this area.  We identified two recent reviews which focused on the effectiveness of interventions to reduce pesticide exposure among farmers. Both of these reviews focused only on the effectiveness of educational interventions. So, there is limited evidence to support the effectiveness of several types of interventions that are used to improve knowledge, attitude, beliefs, practice/ behavior, and exposure monitoring methods related to reducing pesticide exposure among farmers and farmworkers. Also, neither review assessed the methodological quality of the included studies.  (Page 3 and 4, the Introduction section) |
| Objectives | 4 | Provide an explicit statement of questions being addressed with reference to participants, interventions, comparisons, outcomes, and study design (PICOS). | To identify and describe the effectiveness of various types of interventions that have been designed to promote pesticide safety and reduce exposure to pesticides amongst the farmers and farmworkers. We reviewed both RCTs and quasi-experimental interventions. (Page 4 and 5, last paragraph of the introduction section ) |
| **METHODS** | | |  |
| Protocol and registration | 5 | Indicate if a review protocol exists, if and where it can be accessed (e.g., Web address), and, if available, provide registration information including registration number. | The protocol of this review has been approved by the Research and Technology Deputy of Hamadan University of Medical Sciences (ID number: 9410015234). |
| Eligibility criteria | 6 | Specify study characteristics (e.g., PICOS, length of follow-up) and report characteristics (e.g., years considered, language, publication status) used as criteria for eligibility, giving rationale. | We included studies published in English peer-reviewed journals and had the following criteria:  Study Population: Our focus was on farmers and farmworkers, although we did not exclude interventions that had some components for other groups including farmers and farmworkers' families.  Types of studies: We included studies that were randomized controlled interventions (RCT), pretest/ post-test interventions (PPI) or controlled pretest/post-test interventions (cPPI). Descriptive, qualitative, review, systematic review, and meta-analysis studies to reduce exposure to pesticides in agriculture were excluded.  Types of interventions: We included interventions at the national, regional, organizational, community, or individual level deliberately designed to reduce exposure to pesticides or poisoning.  Types of Outcomes: We selected studies in which the primary outcome measure was a subjective (e.g., knowledge/ awareness, attitude, risk perception, and reporting personal protective equipment use) or objective (i.e., measures such as cholinesterase determined by biological monitoring or exposure monitoring) measure of pesticide exposure. We did not consider studies in which the evaluation of outcomes was conducted using qualitative data. In addition, we considered the studies the outcomes of which emphasized only pesticide exposure in three aspects including knowledge, attitude and beliefs, practice or behavior, and exposure monitoring methods.  (Page 5, the Methods section) |
| Information sources | 7 | Describe all information sources (e.g., databases with dates of coverage, contact with study authors to identify additional studies) in the search and date last searched. | We used search terms that covered the concepts of pesticide exposure, farmers and farmworkers, and study design to identify studies in the electronic databases. Search strategies were obtained from the previous systematic reviews. We then modified it to fit the features of the databases. All databases were searched up to **August** 2019.  We searched the following electronic literature databases: PubMed, Agricola, Embase, ISI Web of Science, Science Direct, Scopus, NIOSHTIC-2, Agris. We also tracked the citation of both forward and backward reference lists of included articles and relevant reviews were checked for all included studies.  (Page 4, the Methods section) |
| Search | 8 | Present full electronic search strategy for at least one database, including any limits used, such that it could be repeated. | Search strategy for PubMed  Agricultur* OR agriculture OR farm OR farms OR farmer* OR farming OR "farm worker" OR "farm workers" OR "farmworker" OR "farmworkers" OR ranch* OR dairy OR dairying OR dairies OR greenhous* OR orchard* OR livestock* OR "live stock" OR "live stocks" OR "animal confinement" OR "farm animal" OR "crop production" OR harvesting OR horticultur* OR horticulture OR agronom* OR mix* OR cattle OR animals OR work OR occupation OR gardening OR "crops, agricultural" OR "agricultural workers' diseases" OR gardener OR "agricultural worker" OR "fruit grower" OR orchardist OR grower OR cultivator OR planter  AND  Pesticid* OR pesticides OR fungicid* OR herbicid* OR organophosphate OR biocides OR herbicide OR insecticid* OR carbamat* OR carbamates OR pyrethrins OR molluscacid* OR rodenticid* OR poison* OR "pesticide exposure" OR fungicide OR fumigant  AND  Intervention OR effect* OR "personal protective equipment" OR "safety behavior" OR safety OR prevention OR glove OR mask OR boots OR control* OR protect OR evaluation* OR program* OR training OR "educational program"  AND  Trial OR "randomized controlled trial" OR "quasi-randomized controlled trials" OR "quasi-randomized controlled trial" OR "cluster-randomized controlled trial" OR "cluster-randomized controlled trials" OR "controlled clinical trial" OR "randomized controlled trials" OR "random allocation" OR "double-blind method" OR "single-blind method" OR "clinical trial" OR "clinical trials" OR singl* OR doubl* OR trebl* OR tripl* OR mask* OR blind* OR "latin square" OR placebos OR placebo* OR random* OR "research design" OR "comparative study" OR "evaluation studies" OR "follow up studies" OR "prospective studies" OR "cross-over studies" OR control* OR prospectiv* OR volunteer* OR human NOT animal  (Appendix A) |
| Study selection | 9 | State the process for selecting studies (i.e., screening, eligibility, included in systematic review, and, if applicable, included in the meta-analysis). | Titles and abstracts of studies retrieved by electronic searching were independently screened for eligibility by the three co-authors (MA, FB, and SK). Any discrepancies between the reviewers were resolved through discussion with a fourth review (A. K-Sh). When it was not possible to assess the eligibility of studies based on the title and abstract, full-text versions were obtained.  (Page 6, the methods section) |
| Data collection process | 10 | Describe method of data extraction from reports (e.g., piloted forms, independently, in duplicate) and any processes for obtaining and confirming data from investigators. | Data were extracted independently by two authors (SK and MA). A data extraction template was developed by the research team based on the goals of the study that included the title and author(s), place of study, objective of the study, sample size, characteristics of participants, intervention package (including strategies used, target outcome(s), use of behavioral theory, follow-up duration, measurement tools used to evaluate the effectiveness of intervention), and change in target outcome(s). Any disagreements were resolved by a fourth reviewer (A. K-Sh).  (Page 6, the Methods section) |
| Data items | 11 | List and define all variables for which data were sought (e.g., PICOS, funding sources) and any assumptions and simplifications made. | -- |
| Risk of bias in individual studies | 12 | Describe methods used for assessing risk of bias of individual studies (including specification of whether this was done at the study or outcome level), and how this information is to be used in any data synthesis. | The quality of included studies was assessed by two independent reviewers (MA and SK) using the Effective Public Health Practice Project quality assessment tool (EPHPP). This tool is applicable across multiple study designs. EPHPP rates studies as strong, moderate, and weak in terms of their data collection methods, confounders, study design, selection bias, blinding, dropouts, and intervention integrity. Any discrepancy between two reviewers on the quality rating process was resolved by discussion or by a third reviewer (A. K-Sh).  (Page 6, the Methods section) |
| Summary measures | 13 | State the principal summary measures (e.g., risk ratio, difference in means). | Details of the included studies are reported in Tables 1 to 3. Due to the differences in interventions and target outcomes, no meta-analysis was attempted on the included studies.  (Page 7, the Methods section) |
| Synthesis of results | 14 | Describe the methods of handling data and combining results of studies, if done, including measures of consistency (e.g., I^2^) for each meta-analysis. | -- |

Page 1 of 2

| **Section/topic** | **#** | **Checklist item** | **Reported on page #** |
| --- | --- | --- | --- |
| Risk of bias across studies | 15 | Specify any assessment of risk of bias that may affect the cumulative evidence (e.g., publication bias, selective reporting within studies). | Inter-rater reliability was approved by calculating the percentage of conformity and a Cohen’s Kappa coefficient. We did not exclude studies based on the results of quality assessment. Inter-rater agreement varied across EPHPP component ratings. Overall, there was a good agreement between the two reviewers (Kappa coefficient ranged from 0.66 to 1.00).  (Pages 6 and 7, the Methods section) |
| Additional analyses | 16 | Describe methods of additional analyses (e.g., sensitivity or subgroup analyses, meta-regression), if done, indicating which were pre-specified. | -- |
| **RESULTS** | | |  |
| Study selection | 17 | Give numbers of studies screened, assessed for eligibility, and included in the review, with reasons for exclusions at each stage, ideally with a flow diagram. | A total of 47912 references were identified and reviewed. From these references: 1222 were selected for abstract review and after in-depth abstract review, and 54 were selected for detailed review. Then reference lists of these articles were checked and 14 new references were identified. Finally, we included 32 articles from 31 studies in this review that satisfied our inclusion criteria (Fig. 1). A summary of the included articles is provided in Tables 1, 2 and 3. Results of the Quandt et al study (2013) were reported in the article by Grzywacz et al (2013). Therefore, Quandt et al was considered as the main study. The results of Farahat et al study (2008) were reported in Farahat et al study (2009) and Farahat et al study (2009) was considered as the main study. Thus, two articles came out. Thompson et al. (2008) and Strong et al (2009) reported the results of a study in the form of two articles.  (Page 7, the Results section) |
| Study characteristics | 18 | For each study, present characteristics for which data were extracted (e.g., study size, PICOS, follow-up period) and provide the citations. | Design of the studies: Of the 31 studies, seven studies were controlled pretest/post-test (cPPI) interventions, fifteen studies were pretest/post-test interventions (PPI), and nine studies were randomized controlled trials (RCT).  Study time and settings: Fifteen articles were published in 2010 or later. Fourteen studies were carried out in the USA. The other eighteen studies were conducted outside the USA: four in Thailand, two from Sri Lanka, two from Ecuador, two from Egypt, two from Nepal and one from India, Iran, Australia, Bolivia, and Nicaragua.  Participants and follow-up duration: Most studies had a small sample size. The number of participants in twenty-two studies was less than 200. Sixteen studies focused on farmers/ farmers and their families. Sixteen studies were conducted among farmworkers/ farmworkers and their families. The follow-up duration for studies was often quite short, one month or less in eight studies, less than 6 months in eight studies, and more than 6 months in ten studies. Follow- up in five studies was unknown.  Theoretical framework usage: Of included studies, only eight (25%) used the behavioral theories. The health belief model (HBM) was the most frequent theoretical framework employed. Other theories included social cognitive theory, observational learning theory, and the theory of reasoned action. One study applied both the health belief model and social cognitive theory. Of these studies, only in two studies, components of theories were measured and theoretical frameworks in the remaining studies were used only to guide the intervention development.  Types of outcome measures: Results of the included articles were mainly based on self-reported data and only in seven articles, objective measures such as exposure monitoring methods (e.g. levels of urinary malathion metabolites in urine, plasma cholinesterase and dislodgeable foliar residue (DFR), neurobehavioral status, and observation of participants’ practices were used to evaluate the effects of interventions. Among these articles, five used both objective and self-reported. Among the articles relying on self-reported data, seven articles targeted at knowledge and attitude/beliefs of participants, twelve articles tested knowledge, attitude/ beliefs as well as behavior/ performance of the participants, and five articles assessed only the behavior/performance of participants.  (Pages 7 to 9, the Results section) |
| Risk of bias within studies | 19 | Present data on risk of bias of each study and, if available, any outcome level assessment (see item 12). | Please see Table 4 |
| Results of individual studies | 20 | For all outcomes considered (benefits or harms), present, for each study: (a) simple summary data for each intervention group (b) effect estimates and confidence intervals, ideally with a forest plot. | Please see Table 1 to 3 |
| Synthesis of results and Risk of bias across studies | 21 | Present results of each meta-analysis done, including confidence intervals and measures of consistency.  Present results of any assessment of risk of bias across studies (see Item 15). | Types of intervention and their effects: Due to the heterogeneity designs and outcome variables of included studies, qualitative and semi-quantitative analyses were used to analyze the results.  In order to categorize the interventions, we used the categories provided by Murphy (1980) and Haddon (1996) which divided interventions into five groups, including education/ behavior change, incentive, engineering/ technology, legislation/ enforcement, or multifaceted programs.  Educational/ behavioral intervention: Educational/ behavioral approach (e.g. training the farmers and their families at workplace or through home visit) was used in twenty-one studies. Five of these studies were RCTs, whereas seven were controlled in pretest/post-test interventions (cPPI), and nine employed pretest/post-test designs without control group (PPI). Ten of these studies focused on farmers/farmers and their families, and eleven studies were conducted among farmworkers/farmworkers and their families. Of the 21 studies that applied educational/ behavioural approach, 14 studies (67%) were assessed as having low quality.  Eighteen studies (including 8 studies among farmers/farmers and their families and 10 studies among farmworkers/ farmworkers and their families) categorized in the educational/ behavioral approach measured the participants' knowledge as an outcome. The result of these studies showed that all but one study conducted among farm workers and their families were successful; however, the quality of about 59% of these interventions was graded low.  All seven studies (including four studies among farmers/farmers and their families and 3 studies among farmworkers/ farmworkers and their families) which measured the participants' attitude/ beliefs were effective. However, four studies were assessed to have low quality.  Participants' behaviour /practices were considered as outcome in fourteen studies (including 9 studies among farmers/farmers and their families and 5 studies among farmworkers/ farmworkers and their families), participants' behaviour /practices were considered as outcome variable. Of these studies three were not successful, and two improved in some assessed behaviour and practices. The quality of about 45% of effective interventions was graded low. Interestingly, while about 89% of studies targeted farmers/ farmers and their families were successful in making a significant change in behavior/ practice, only 20% of studies targeted farmworkers/ farmworkers and their families have been reported as completely effective.  Of educational/ behavioral interventions, only four studies (including two studies among farmers and two studies among farmworkers/ farmworkers and their families) evaluated the effectiveness using objective measures (e.g., biomarkers of pesticide exposure and neurobehavioral test). While of four studies, two conducted among farmers were found effective, none of the two studies conducted among workers were completely effective.  Incentive intervention: Incentive intervention consisted of providing money/PPE or positive feedback. We found only one study that specifically evaluated an incentive intervention. The results revealed that it was not effective in making a change in acute organophosphate poisoning symptoms and plasma cholinesterase.  Engineering and technological interventions: Engineering/technology interventions consisted of improving the pesticide sprayer machines and equipment and replacing hazardous structures with safer ones. We did not find any studies that specifically evaluated only an engineering and technological intervention.  Legislation/enforcement intervention: We did not find any studies that specifically evaluated only a legislation/enforcement intervention.  Multifaceted programs: Multifaceted programs were applied in ten studies (including 5 studies among farmers/farmers and their families and 5 studies among farmworkers/ farmworkers and their families) which included a combination of interventions such as education, home visits, providing personal protective equipment (e.g., coveralls and gloves), laundry service, warm water and soaps, containers for storing work shoes and clothes. Of these studies, four were RCT, and six employed quasi-experimental designs without control group (PPI). Of ten multifaceted programs, nine studies were assessed to have a low-quality.  Of nine studies that used a multifaceted approach, three low-quality studies assessed the participants' knowledge as an outcome, which was effective. In eight studies, participants' behavior/ practices were considered as the outcome. Of these studies four low-quality studies which focused on farmers/ farmers and their families were successful in making change in participants ’behaviors, whereas three studies which focused on farmworkers/ farmworkers and their families improved in some assessed behavior and practices. In three studies, objective measures were used to evaluate the effectiveness of the multifaceted programs: two among farmworkers and one among farmers. Of these studies two were effective in assessed metabolites.  (Pages 9 to 12, the Results section) |
| Additional analysis | 23 | Give results of additional analyses, if done (e.g., sensitivity or subgroup analyses, meta-regression [see Item 16]). | -- |
| **DISCUSSION** | | |  |
| Summary of evidence | 24 | Summarize the main findings including the strength of evidence for each main outcome; consider their relevance to key groups (e.g., healthcare providers, users, and policy makers). | To the best of our knowledge, there are no systematic reviews that examine comprehensively the effectiveness of interventions to promote pesticide safety and reduce pesticide exposure among farmers or farmworkers. Indeed, we conducted this review to address the gaps of previous efforts. The majority of the studies included were quasi-experimental (21/31). This might be due to the affordability and feasibility of this type of design. Moreover, 75% (24/32) of articles were categorized as low quality.  Although we expected to find evidence on the effectiveness of five interventional approaches, we discovered no studies investigating specifically the legislation/ enforcement or engineering/ technology interventions. We also found only one study that specifically addresses the effectiveness of the incentive intervention. However, this type of intervention was used in combination with other types of interventions (i.e., multifaceted intervention). The majority of studies were based specifically on educational/ behavioral interventions. In addition to studies that specifically evaluated educational interventions, this approach has been used in all the multifaceted interventions as a key component. Although the evaluated educational interventions were effective in general, the following two considerations should be taken into account in the interpretation of results: First, the main success of these studies was to change participants' knowledge and beliefs and in cases that practices and objective measures were considered as the outcomes, the interventions were less likely to be successful. Second, the positive results of these interventions should be interpreted in light of the design and quality of studies.  The majority (68.8%) of the studies used this approach were quasi-experimental designs and about 71% were categorized as low-quality studies and only one study was ranked as having a strong quality. This case becomes clear considering that in terms of all outcomes; results of quasi-experimental studies were more successful than the findings of RCTs. This could be related to the inherent weakness of quasi-experimental design for evaluating the effectiveness of alternative intervention rigorously. Similar concerns have been raised about other reviews evaluating the effectiveness of educational interventions among farmers and farmworkers.  Another finding of our review is that compared to farmworkers and their families, the interventions which targeted farmers and their families achieved more success. There are some explanations for this result. Farmworkers may have lower-incomes, lower education levels, cultural /language barriers, and in some cases temporary work permits. Also, they may have less control over the work conditions.  This review highlights the limited research on this field in LMICs. While only 3.15% of total employment in HICs (vs. 36.15 in low-middle income countries) relates to the agriculture sector more than 50% of the identified studies were based on data from HICs. Also, regarding the type of design of the included studies, our review showed that seven out of nine studies which used RCT were conducted in USA which highlights the challenges associated with performing RCT in LMICs.  The multi-faceted intervention would be expected to reveal more positive results than educational/ behavioral interventions. However, our review showed that this type of intervention was partially successful in changing promote pesticide safety and reduce pesticide exposure It should be noted that multifaceted interventions in the included studies consisted of a combination of educational/ behavioral and incentive interventions. In other words, no multi-faceted intervention studies used engineering/ technology or legislation/ enforcement approach as part of their intervention strategy. This might be due to the implementation challenges and limited infrastructure. It seems that to be effective, educational/ behavioral interventions need to be embedded in contextual factors and combined with engineering/ technology or legislation/enforcement approaches. Although there are many laws and regulations to protect farmers and farmworkers from pesticide risks-especially in HICs- investigation on their adaptation, implementation, and evaluation remain underexplored in the literature.  Regardless of study design and type of intervention used, our review showed that the interventions were most likely to be successful in making a change in participants' knowledge/ beliefs. However, it should be noted that only in seven studies (out of 21) that evaluated knowledge/ beliefs, follow up duration was longer than six months. This means that it is not clear that the obtained changes would be sustained over time.  Only eight articles (out of 32) determined objective measures to evaluate the effects of interventions. This may be due to several reasons: lack of skill in evaluation plan, insufficient funding, and supervision, and lack of available resources and equipment.  Regarding outcomes other than knowledge/ beliefs, the success of interventions was partial. We believe that there are at least four explanations for this finding. First, while the increasing evidence suggests that the legislation/enforcement approach had an influence on the effectiveness and sustainability of health-related interventions in other contexts, the findings of this review showed that the majority of studies used only educational/ behavioral approaches.  Health promotion activities can play a significant role in increasing visibility and highlighting the importance of legislation/ enforcement interventions. Given the fact that the effects of the legislation/enforcement or engineering/ technology interventions do not appear immediately, design studies such as time series may be used to evaluate these types of interventions. Second, this may reflect lack of attention to behavior change technique (BCT) according to targeted outcomes. Indeed, it seems that the majority of included studies involved providing the overall information regarding the risks and behavior and did not apply behavioral methods such as goal setting, planning/ implementation, and social encouragement/ support. There is evidence suggesting that providing individually tailored information (vs. overall information and using particular behavior change techniques are associated with more effectiveness. We recommend a review for future focusing on comparisons of the different behavior change methods. Third, it is evident that considering social and behavioral science theories in developing public and health promotion programs is associated with more effectiveness. These frameworks help to understand health behaviors and the contexts (such as cultural, economic, and social circumstances) in which they occur. Our findings revealed that a majority of studies (about71 %) did not explicitly apply social or behavioral theories. Fourth, it should be taken into account that changing the behavior of farmers and farmworkers is difficult and many protective recommendations are never adopted by farmers. Based on the ecological model, for attempts to be effective in farmers' and farmworkers' health and modify farmers/farmworkers' behavior, it is necessary to develop multilevel interventions (i.e., intervention at individual, interpersonal, organizational, community, and public policy level) targeting different barriers. The results of this study and the existent evidence show that most programs in this area tend to focus on the individual farmers/ farmworker and limited attention has been paid to involving the higher layer of the ecological model. In this study, we found that the focus of about 54% of the studies was on the farmers/ farmerworkers, and the rest of the interventions had some components for their families. In this way, the higher layers of ecological model have been ignored.  (Pages 12 to 16, the Discussion section) |
| Limitations | 25 | Discuss limitations at study and outcome level (e.g., risk of bias), and at review-level (e.g., incomplete retrieval of identified research, reporting bias). | This review has limitations. We included multiple types of outcomes and study designs which makes it impossible to perform a meta-analysis. In addition, in order to assess the higher quality evidence of the effectiveness of the intervention, we didn’t search the grey literature; therefore, a publication bias may exist in this review.  (Page 16, the Discussion section) |
| Conclusions | 26 | Provide a general interpretation of the results in the context of other evidence, and implications for future research. | The studies included in this review addressed the effectiveness of interventions for farmers/ farmworkers to promote pesticide safety and reduce pesticide exposure. The majority of studies relied on only education/ behavior change. We also found some studies that reported the effects of multifaceted programs combining more than one approach such as education, home visits, and providing personal protective equipment. Although the interventions were effective in general, the results should be interpreted in the light of design limitations and self-reported outcomes. Further research is crucial to understand the role of other than educational/ behavioral interventions and the effectiveness of well-designed on more objective outcomes.  (Pages 16 and 17, the Conclusion section) |
| **FUNDING** | | |  |
| Funding | 27 | Describe sources of funding for the systematic review and other support (e.g., supply of data); role of funders for the systematic review. | This work was supported by Hamadan University of Medical Sciences [reference number: 9410015234]. |

*From:*  Moher D, Liberati A, Tetzlaff J, Altman DG, The PRISMA Group (2009). Preferred Reporting Items for Systematic Reviews and Meta-Analyses: The PRISMA Statement. PLoS Med 6(7): e1000097. doi:10.1371/journal.pmed1000097

For more information, visit: **www.prisma-statement.org**.
